# Learning and Timing of Voluntary Blink Responses Match Eyeblink Conditioning

**DOI:** 10.1038/s41598-017-03343-2

**Published:** 2017-06-13

**Authors:** Anders Rasmussen, Dan-Anders Jirenhed

**Affiliations:** 1 Department of Experimental Medical Science, Lund University, BMC F10, 22184 Lund, Sweden; 2 Department of Neuroscience, Erasmus Medical Center, 3000, Rotterdam, The Netherlands

## Abstract

Can humans produce well-timed blink responses to a neutral stimulus voluntarily, without receiving any blink-eliciting, unconditional, stimulus? And if they can, to what degree does classical eyeblink conditioning depend on volition? Here we show that voluntary blink responses learned in two paradigms that did not involve any unconditional blink-eliciting stimuli, display timing that is as good, or better than, the timing of blink responses learned in a standard eyeblink conditioning paradigm. The exceptional timing accuracy likely stems from the fact that, in contrast to previous studies, we challenged our participants to blink in a timed manner, and not merely to blink so as to avoid the corneal air puff. These results reveal a remarkable level of voluntary control over a simple movement, and they challenge the view that learning during eyeblink conditioning is necessarily automatic and involuntary.

## Introduction

The ability to time behavior and estimate temporal intervals are fundamental skills that depend on the cerebellum^1–3^. Eyeblink conditioning, in which participants learn to produce adaptively timed blink responses, has been one of the most popular paradigms for studying associative and timing dependent learning^4^. In eyeblink conditioning, a subject is presented with a neutral conditional stimulus (CS), usually a tone, followed by a blink-eliciting, unconditional stimulus (US), such as an air puff to the cornea. After some repetitions, the subject acquires a conditioned blink response (CR), with an onset before and a peak matching the expected CS-US interval^4^. Being categorized as a form of non-declarative motor learning, eyeblink conditioning should not depend on awareness of stimulus contingencies^5^. In support of this view, amnesic patients who are unaware of the relation between the CS and the US, perform as well as non-amnesic controls^6^. However, individuals who understand the stimulus contingencies outperform those who do not grasp the CS-US connection^7^. This finding is consistent with other studies that also indicate that awareness influences learning in eyeblink conditioning^8–10^.

Awareness might influence eyeblink conditioning by increasing the number of voluntary responses the subject makes when hearing the tone. Despite efforts to ascertain the role of volition in eyeblink conditioning, the issue remains unsolved^5, 11, 12^. Early studies tried to prevent voluntary responses by instructing participants not to force nor to inhibit their blinks^12, 13^. However, it cannot be objectively verified that such instructions are obeyed.

In subsequent studies, the onset latency and the shape of the blink responses were used to weed out voluntary responses^14^. The latency method involved excluding participants who had a high proportion of blinks with an early onset^15, 16^. This procedure was, however, deemed unreliable in a follow-up study because it only identified a fraction of the voluntary responders. Instead, the authors proposed that voluntary blinks are associated with faster eye closure, resulting in a steeper slope^17^. Yet, when the slope and latency criterion were compared in a comprehensive study, it was found that the two methods result in different outcomes and that both have serious deficiencies^18^. This is where things still stand.

## Methods

### Subjects and procedure

Twenty-six students enrolled in the medical program at Lund University were recruited to the study. The first 16 participants were trained with visual feedback. Both online observations and the offline analysis revealed very clear effects so for the follow up experiment, we only tested 10 participants. Of all 26 participants, five were excluded because the quality of the signal was not good enough to ensure accurate estimation of the onset and peak of the blink responses. This study was carried out in accordance with the ethical guidelines described in the Belmont report and the declaration of Helsinki. The regional ethics committee in Lund, Sweden, approved this study and the associated procedures.

Informed consent was obtained from each participant before the experiment began. The participants were told that they would hear a tone and that they should try and blink with a specific delay after the tone. Of the 21 participants, 13 were told that they would see the recording of their eyelid movement following the most recent tone, along with a marker indicating the timing target, i.e., the delay with which they should try to blink. Importantly, the eyelid trace was not shown until after the end of the trial, meaning that the subjects could not use the information on the screen to time their blinks during the current trial, but only to guide whether to blink earlier or later on the following trial(s). To ensure that all subjects understood what they were supposed to do and that they understood the information on the screen, the participants received verbal feedback from the experimenter on the first few trials.

The remaining eight participants were challenged to blink “just before” a click sound, that did not itself trigger a blink response. The click sound was generated by the opening of the gas valves that would normally allow gas to flow from the pressurized tube, containing nitrogen gas (N^2^), to produce a brief air puff to the eye. But since we had disconnected the gas tube, the opening of the valves only resulted in a gentle click sound. In other words, the stimuli presented were identical to those presented during standard eyeblink conditioning, with the crucial difference that here the subjects did not receive any air puffs. Again, to ensure that the subjects understood the task, the experimenter provided verbal feedback after the first few trials.

**Figure 1:**
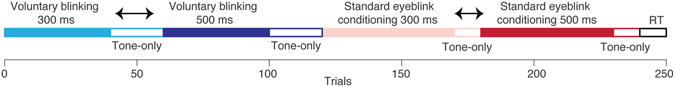
Experimental protocol. First, participants were trained to produce accurately timed blinks voluntarily (light and dark blue) in response to a tone, using visual feedback or a click sound. The target time was first 300 ms and then 500 ms, or vice versa. After each voluntary training session (40 trials), the participants were instructed to continue to blink to the tone with the same delay, but without the feedback. Next, participants were trained in a standard eyeblink conditioning protocol (light and dark red) with the same two intervals. Training consisted of 50 trials with 20 percent probes, followed by 10 tone only trials. In the last ten trials participants were instructed to blink as rapidly as possible.

All participants were trained to blink to two different timing targets, 300 and 500 ms, the order of which was randomized across all participants. Following 40 trials in which participants saw their blink trace or heard the click, the feedback was discontinued by turning off the screen or the click sound. The participants were challenged to continue to blink with the same delay, without the feedback. After 20 trials with no feedback, we switched to the second timing target and presented 40 trials with feedback followed by another 20 trials without feedback (Fig. 1).

Having completed training to two different time intervals in one of the two paradigms described above, all participants were trained in a standard eyeblink conditioning paradigm, with the same two intervals, 300 and 500 ms. We instructed the participants to try to be passive while they watched a silent movie (Charlie Chaplin). The training consisted of 50 paired trials in which the tone was followed by an air puff delivered to the cornea. Each trial had a 20 percent chance of being a probe trial, that is a trial without an air puff. At the end of these 50 trials, we presented an additional 10 CS-alone trials (no air puff). After these 60 trials (50 paired and 10 CS-alone trials), we switched to the second timing target and ran the same protocol again.

After training in one of the two voluntary paradigms (visual feedback or click sound) and after completing the standard eyeblink conditioning protocol, we tested the participants blink reaction time by challenging them to blink as fast as possible after they heard the tone. This was done to check if the blink responses in the other paradigms were indeed timed, and to ensure that the participants were not merely blinking when they heard the tone. When these five sub-tests had been completed, which took approximately one hour, the participant was thanked for their participation. As a token of our gratitude for their participation they received one movie voucher.

### Equipment and stimuli

To trigger stimuli and record eyelid movements we used a Micro 1401- AD converter (Cambridge Electronic Design, CED), connected via USB to a PC running Windows 7. Spike2 v7 software (CED) was used to acquire da﻿ta from and program the micro 1401. The 1000 Hz, 1 s tone that served as a CS was transmitted by the DAC port on the Micro 1401, to PC loudspeakers. We always made sure the participant heard the tone clearly, and that it did not trigger a blink response (startle response). The click sound and the air puff were generated by a D132202 solenoid valve (AirCom), that was triggered by one of the digital ports on the Micro 1401. To deliver the air puff, we connected a tube containing pressurized nitrogen gas (N^2^) to the solenoid valve. To record eyelid movements, we placed a round neodymium magnet (diameter = 4 mm, thickness = 1 mm), on the eyelid of the participant, using double cohesive tape. If the magnet prevented normal eyelid movements, we switched to a smaller magnet (diameter = 3 mm). The participants were then asked to put on a pair of custom made glasses, equipped with a GMR sensor (NVE Corporation), that measured the location of the magnet, as well as a nozzle that could direct the air puff onto the cornea.

### Analysis

Stimulus triggers and eyelid movements, sampled at 2000 Hz, were recorded, unfiltered, in Spike2 v7. All subsequent analysis was done with custom made scripts in Matlab (MathWorks). For each trial, we determined if there was a blink response and if so, when the onset and the peak of that response occurred. On trials with an air puff, click or visual feedback, a response was categorized as a learned response, if the onset occurred before the timing target or the air puff, but at least 150 ms after the onset of the tone. On tone-only trials, a response was deemed a CR if the onset was between 150 and 800 ms after the onset of the tone. All response categorizations made by our script were subsequently checked and corrected if obvious errors were present, which occurred in less than 10% of the trials. Group differences were tested using pairwise or independent two-tailed t-tests. All tests on the timing of blink responses was based exclusively on CS alone trials, with no US and no feedback. For each comparison, we also calculated Cohen’s d effect size and 95% confidence intervals. The datasets and analysis files generated during the current study are available from the corresponding author on reasonable request.

## Results

**Figure 2:**
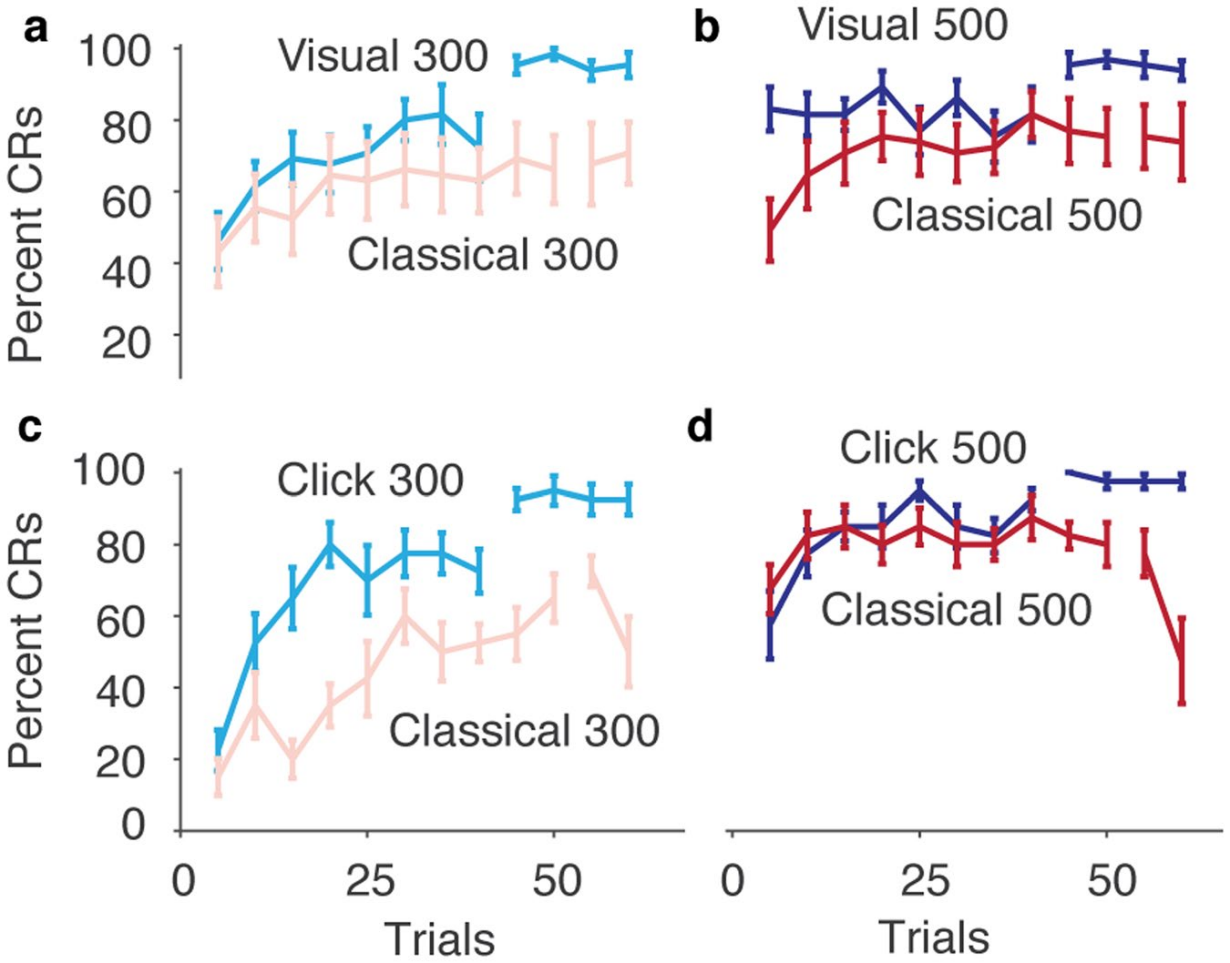
Learning curves. (**a**–**d**) Percentage of blink responses in successive blocks of five trials, that would be categorized as conditioned responses (CRs) according to standard criteria (a blink starting later than 150 ms after tone onset and before the target time or time of the air puff). The break in the lines indicate where Click or Visual feedback was ceased (**a** and **c**), or when the air puff was no longer delivered (**b** and **d**).

In both voluntary paradigms, participants quickly learned to produce accurately timed blink responses without receiving any unconditional stimuli. Using standard criteria for the identification of conditioned responses (an onset >150 ms after the CS onset and before the target time), we found that the percentage of adaptively timed responses rose as fast or faster in the two voluntary paradigms compared to the standard eyeblink paradigm (Fig. 2). This was the case despite the fact that the voluntary paradigm always preceded the classical paradigm.

**Figure 3:**
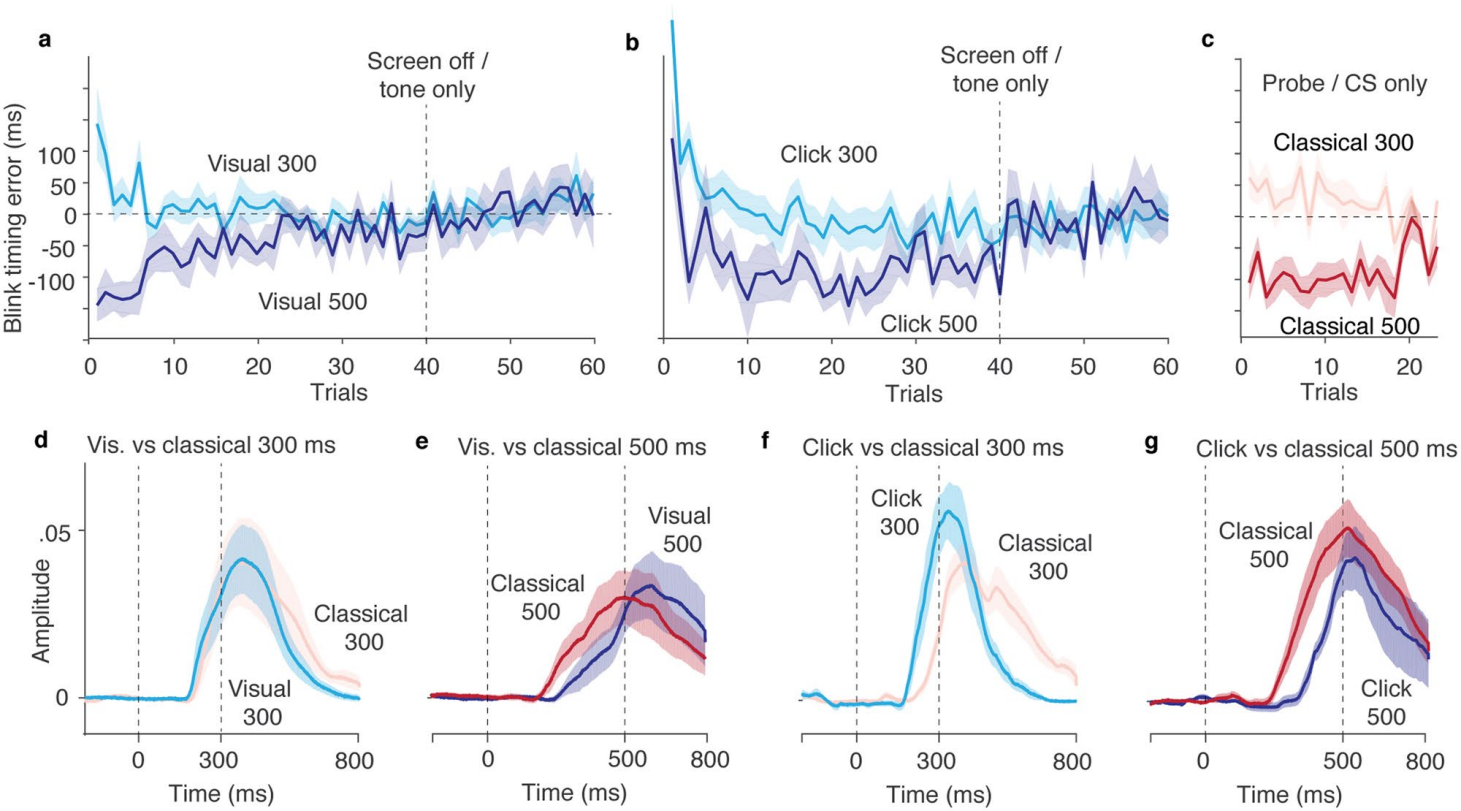
Timing of blink responses. (**a**,**b**) Learning of voluntary response timing. Average (mean ± SEM) difference between the target time and the peak of the blink response for the delayed visual feedback paradigm and the click paradigm. (**c**) Response timing (mean ± SEM) on randomly interspersed probe trials during training and on the 10 CS alone trials after training in the standard eyeblink. (**d**–**g**) Comparison of blink responses on CS alone trials after training in a voluntary paradigm (dark and light blue) and a standard eyeblink conditioning paradigm (red and pink).

As expected, given that it was a novel task, the participants initially blinked too early or too late. However, in both voluntary paradigms, irrespective of the delay used, the participants rapidly learned to blink with an onset just before, and a peak very close to, the target time. Even when feedback was no longer given, the participants continued to produce accurately timed blink responses, indistinguishable from conditioned responses (Fig. 3). When we switched to the standard eyeblink conditioning paradigm, we observed a similar convergence of the timing of the peak responses (Fig. 3c). With a 300 ms ISI, peaks occurred after the expected US while a 500 ms ISI resulted in peaks that occurred before the expected US. Because the US masks late CRs, we could only use the 20% randomly interspersed probe trials and the CS alone trials to analyze timing changes during standard eyeblink conditioning.

**Figure 4:**
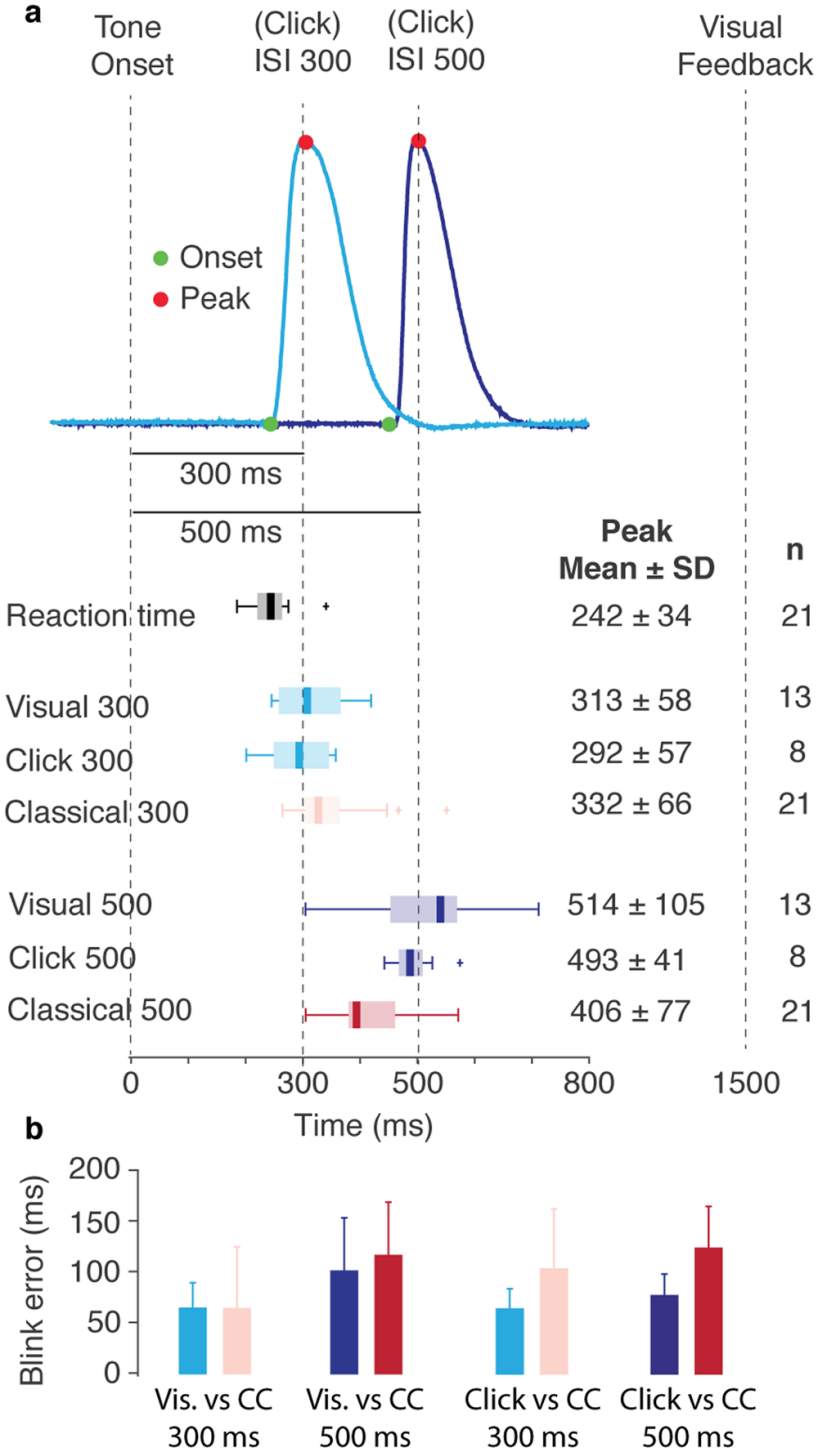
Timing comparisons. (**a**) Top: Two blink responses produced after training in the click paradigm with two different delays, 300 ms (light-blue) and 500 ms (dark-blue). Green and red markers denote the identified onset and peak respectively. Bottom: Boxplots illustrating the timing of the peak in the different paradigms. (**b**) The timing error variability on CS alone trials for the different training paradigms and target times.

Statistical tests verified that the timing of the CR peak depended on the target time, in both voluntary paradigms (Fig. 4A). Participants who received visual feedback blinked later when the target time was 500 ms compared to when the target time was 300 ms, t(12) = 9.3, p < 0.0001, d = 2.4, CI = [249, 154]. Similarly, participants who were challenged to blink before the click sound also blinked later when the click came after 500 ms compared to when it came after only 300 ms, t(7) = 8.0, p < 0.0001, d = 4.1, CI = [260, 141]. Moreover, blinks on trials with a 300 ms target time had longer latencies than blinks on reaction time trials. This was the case for those who received visual feedback, t(12) = 5.2, p = 0.0002, d = 1.5, CI = [43, 105], as well as for those who blinked before the click sound, t(7) = 3.1, p = 0.016, d = 1.0, CI = [11, 80]. Indeed, of the 21 participants, 19 had longer delays to the onset of the blink responses on the 300 ms target time trials compared to the reaction time trials. The fact that participants blinked with different delays to different target times show that the voluntary blink responses were adaptively timed.

There was no difference in the timing of blink responses between the two voluntary paradigms. Thus, participants blinked with the same delay when the target time was 300 ms, irrespective of whether they received visual or auditory feedback, t(19) = 0.80, p = 0.43, d = 0.36, CI = [−33, 75]. Likewise, there was no difference between the two paradigms when the target time was 500 ms, t(19) = 0.54, p = 0.60, d = 0.26, CI = [−61, 103]. This demonstrates that the two voluntary paradigms produce comparable voluntary blink responses.

There were some differences between the timing of the learned blink responses in the voluntary paradigms compared to the standard eyeblink conditioning paradigm. The timing of blink responses following training with visual feedback, with a 300 ms target time, was marginally different from the timing of blink responses following eyeblink conditioning with a 300 ms ISI, t(11) = −0.66, p = 0.52, d = −0.22, CI = [−54, 29]. Yet, when the target time or ISI was 500 ms, the voluntary blink responses were associated with a significantly longer delay, t(12) = 4.83, p = 0.0004, d = 1.04, CI = [52, 137.621]. A similar pattern was found when participants were challenged to blink before a click sound. For the 300 ms target time or ISI, the voluntary responses had a shorter delay than the responses acquired in the standard eyeblink conditioning paradigm, t(7) = −3.05, p = 0.0185, d = −1.60, CI = [−170, −21.6]. Conversely, when the target time or ISI was 500 ms, the voluntary blink responses were associated with marginally longer delay, t(7) = 2.31, p = 0.0546, d = 1.23, CI = [−1.9, 152].

We also examined the timing precision of blink responses by calculating the time difference between the peak of the blink responses and the target time or the ISI (Fig. 4B). This analysis revealed that voluntary blink responses were as precise, and in one case more precisely timed than the blink responses acquired during standard eyeblink conditioning. Specifically, participants who were challenged to blink before a click sound with a 500 ms target time, had a smaller average error compared to when they were trained in a standard eyeblink conditioning paradigm with a 500 ms ISI, t(7) = −4.08, p = 0.0046, d = −1.45, CI = [−73, −19]. For the other comparisons there were no significant difference between the voluntary and the classically conditioned blink responses (p > 0.15).

## Discussion

Our results show that, contrary to popular beliefs, humans can learn to produce adaptively timed blink responses voluntary, without receiving a single reflex eliciting unconditional stimulus. The timing of these voluntary blink responses is retained even when participants no longer receive any feedback. To our knowledge, no prior studies have demonstrated the same level of precision in the timing of voluntary blink responses. This, we believe, is because we explicitly challenged our participants to time their blinks. Earlier studies, by contrast, have asked participants to blink so as to avoid the air puff^19, 20^, which can be accomplished by blinking immediately after the tone. This strategy would result in blinks with a short onset latency, which is exactly what has been observed^21^. In other words, the timing of voluntary blink responses in prior studies may have been poorer simply because participants were not required to time their blinks.

Did volition play a role in the standard eyeblink conditioning paradigm in our study? The fact that the rate of adaptively timed blink responses dropped from nearly 100%, in the end of the voluntary session, to 20–60% in the beginning of the conditioning session suggests that the participants were, at least to a certain degree, following our instructions to be passive. However, this drop does not prove that the timed blink responses in the eyeblink conditioning paradigm were truly conditioned. A rate of 20–60% CRs is still high for the fist five trials. And even though the participants claimed that they did not blink voluntarily, they may not know if their behavior was automatic or voluntary. In this and other studies, it is conceivable that the mechanisms that allow participants to blink voluntarily with precise timing also contribute to learning during standard eyeblink conditioning.

Our results thus emphasize the need for new ways to determine if blink responses, acquired in a standard eyeblink conditioning paradigm, are classically conditioned automatic blink responses, or if they are partly or entirely voluntary. Previous reports suggest that voluntary blink responses have a shorter onset latency^15^. This was not the case here. The timing of voluntary blink responses was as good or better than the timing of blink responses learned in the standard eyeblink conditioning paradigm. Thus, as others have argued^18^, the onset latency, like the slope, is an unreliable criterion for separating voluntary and classically conditioned blink responses. Indeed, voluntary contributions might explain why humans only need around 50 trials to acquire a high rate of CRs^22, 23^, while other species often need several hundred trials^4, 24^.

Our results do not address the neural mechanisms that generate the adaptively timed voluntary blink responses. There is convincing evidence that the cerebellum is necessary for eyeblink conditioning in animals^25–27^. The evidence from human subjects, likewise, suggest that the cerebellum is involved in eyeblink conditioning^23, 28, 29^. It has been argued that the cerebellum is important for learning all kinds of temporal relationships in the millisecond range^30^. If that is the case, then adaptively timed voluntary blink responses presumably also require the cerebellum. On the other hand, voluntary behaviors are almost universally believed to be initiated in the cerebral cortex. Future studies should examine the relative contribution of the cerebellum and the cerebral cortex to the generation of adaptively timed blink responses.
